# Application of Microfracture Analysis to Fatigue Fractures in Materials through Non-Destructive Tests

**DOI:** 10.3390/ma17040772

**Published:** 2024-02-06

**Authors:** Ulises Sánchez-Santana, Gerardo Presbítero-Espinosa, José María Quiroga-Arias

**Affiliations:** 1 Centro de Ingeniería y Desarrollo Industrial, Pie de la Cuesta 702, Desarrollo San Pablo, Querétaro 76130, Mexico; 2 Universidad Aeronáutica en Querétaro, Querétaro 76278, Mexico; chema1807@gmail.com

**Keywords:** microfracture, image processing, neural network, simulation analyses

## Abstract

Fatigue fractures in materials are the main cause of approximately 80% of all material failures, and it is believed that such failures can be predicted and mathematically calculated in a reliable manner. It is possible to establish prediction modalities in cases of fatigue fractures according to three fundamental variables in fatigue, such as volume, number of fracture cycles, as well as applied stress, with the integration of Weibull constants (length characteristic). In this investigation, mechanical fatigue tests were carried out on specimens smaller than 4 mm^2^, made of different industrial materials. Their subsequent analysis was performed through precision computed tomography, in search for microfractures. The measurement of these microfractures, along with their metrics and classifications, was recorded. A convolutional neural network trained with deep learning was used to achieve the detection of microfractures in image processing. The detection of microfractures in images with resolutions of 480 × 854 or 960 × 960 pixels is the primary objective of this network, and its accuracy is above 95%. Images that have microfractures and those without are classified using the network. Subsequently, by means of image processing, the microfracture is isolated. Finally, the images containing this feature are interpreted using image processing to obtain their area, perimeter, characteristic length, circularity, orientation, and microfracture-type metrics. All values are obtained in pixels and converted to metric units (μm) through a conversion factor based on image resolution. The growth of microfractures will be used to define trends in the development of fatigue fractures through the studies presented.

## 1. Introduction

A fatigue fracture is defined as a “partial or complete fracture due to its inability to withstand non-visible stresses applied rhythmically, repeatedly, and below the threshold” [1] Fatigue fractures occur due to the accumulation of stress-induced microfractures. One should investigate the stress intensity factor and the outcomes of both experimental (analytical) and numerical calculations for the range of stress intensities in order to evaluate the effects of cyclic fatigue on metallic specimens with diverse physical features [2]. Using heterogeneous image fusion to reduce picture-related disturbances in intensity or range image data and to reduce uncertainties through cross-domain feature correlation, a deep convolutional neural network-based crack segmentation methodology was provided [3]. For automatic quality control, a unique deep learning model was created; it integrates both local and global context information by using per-pixel segmentation and per-image categorization [4]. For automatic quality control, a unique deep learning model was created, which integrates both local and global context information by using per-pixel segmentation and per-image categorization applied to microCT images [5]. On the other hand, fresh studies on bone tissue have improved our comprehension of fracture mechanics [6,7].

They begin as microfractures and become more extensive with repetitive stresses, until reaching a macrofracture size of approximately 1 mm, the size necessary to result in a true fracture through the structure of a material [8]. Weibull analysis is the most commonly chosen technique for estimating a probability based on measured or assumed data. The Weibull model has an interesting property linked to the fact that, depending on the values of α, it can present increasing, decreasing, or constant failure rates. Thus, when α = 1, the Weibull model becomes exponential and has a constant failure rate. The exponential model is therefore a particular case of the Weibull model. When α > 1, the model has an increasing failure rate, and when α < 1, it has a diminished failure rate. The Weibull model is very versatile, and, in practice, it is one of the most used. For the two-parameter Weibull distribution, the relationship between the cumulative probability *P* and fracture length α is given by Equation (1), where a0 and α are constants.

(1) $$P=1-e^{{-\left(a/a_{0}\right)}^{\alpha }}$$

(2) $$\ln\left[-\ln\left(1-P\right)\right]=\alpha \left[\ln\left(a\right)-\ln\left(a_{0}\right)\right]$$

To describe and present the data in a more understandable way, Equation (1) undergoes linearization, which is presented in Equation (2). In this way, the data obtained can be graphed and interpreted.

Fatigue fractures involve approximately 80% of all material failures [9]. In human bones, fatigue fractures often occur in military recruits, with 0.91% of male recruits and 1.09% of female recruits suffering fatigue fractures. Between 4.7% and 15.6% of all runners’ injuries are stress fractures [10]. Deep learning in neural networks can detect fractures in materials with up to 98% accuracy, according to recent research [11]. In the same way, image processing can be used to detect gradients, which are analyzed to find the best value [12]. In a recent study [13], the authors compared the detection of cracks in concrete walls using both methods. The TernausNet-based deep learning method was found to be more accurate, at 81.9% accuracy, while the threshold-change method was more accurate, at 80.3% [14].

The primary goal of this project is to identify, establish, and validate methodologies and research areas toward accurate prediction processes for the prevention of fatigue fractures in industrial materials through the creation of microfractures and the application of non-destructive testing. Although there have been studies in the literature on the examination of microfractures in various materials, there is still much to be learned about how microfractures originate, spread, and ultimately induce material fracture in each individual situation. As a result of the external fatigue loads given to the material in each unique example under investigation and analysis, we have developed a system for studying microfractures, which, we believe, may be quantified and compared with other materials, whether the same or similar, in relation to their creation and growth.

Our studies have been implemented mainly in cortical bone structures, although it is important to determine that bone is a composite material, and this methodology can be executed in the analysis of the development of microfractures in different types of industrial materials [15], such as those in the aerospace industry, metallurgy, composite materials, historical buildings, monuments, as well as earthquakes, among others. With the integration of the Weibull distribution constants of two parameters (characteristic length), prediction modalities can be applied in cases of fatigue fracture based on three basic fatigue variables: volume, number of cycles to fracture, and applied stress. Therefore, the implementation of a broad area of study and innovation is studied, which will be based on the performance of mechanical tests through the application of fatigue stress and the characterization of properties in materials through non-destructive testing. This aims at the prevention, comparison, and evaluation of fractures in different types of industrial materials due to fatigue.

In this study, we developed a computer mesoscale model of bone tissue fatigue fractures, taking into account the formation, coalescence, and interaction of pre-existing micro-fractures with the microstructure. Using diverse commercial software, the microstructure of bone tissue was modeled from computed tomography and scanning electron microscopy data.

## 2. Methodology

To establish comparisons in the determination of Weibull constants, specimens measuring 2 mm × 2 mm × 3 mm were made. In order to establish compression tests under fatigue, we used an MTS landmark universal testing machine. Cyclic axial compression forces were applied to the samples, at a frequency of 3 Hz with an applied stress of 80 MPa, with cycles between 10 and 100 MPa. This constant stress range was applied until fracture occurred. Once the fatigue tests were developed, the specimens were analyzed using X-ray computed tomography (Phoenix v|tome|x m), at the facilities of the Center for Industrial Engineering and Development (CIDESI), which is affiliated to the National Council of Humanities, Science and Technology (CONAHCYT) in Mexico. Microfractures were identified and isolated from the rest of the reconstructed specimen volume to proceed with the calculation of the corresponding Weibull constants.

**Figure 1:**
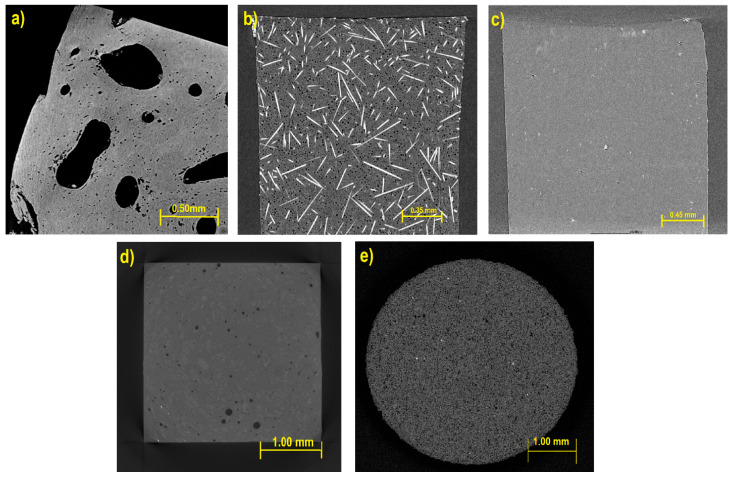
Computed tomography of specimens of (**a**) bone tissue; (**b**) nylon with 5% hollow microspheres; (**c**) Sigma food material; (**d**) ultra-high molecular weight polyethylene and (**e**) granite.

Therefore, through these fatigue tests and the use of the study of microfracture accumulation, based on the concept of characteristic length within the Weibull distribution, the implementation of new methodologies will be allowed. This includes exploring new areas of study within the field of fracture mechanics in materials, based on precise models aimed at preventing the development of fractures of industrial materials due to fatigue. As the first material, there is bone tissue, on which fatigue tests have already been carried out previously in a previous investigation. This material served as the starting point for this research, as it already had computed tomography images (Figure 1) that allowed the neural network to be trained, as well as to be used in the simulation stage. Additionally, for image processing, images of metallic materials, nylon-based materials, ultra-high molecular weight polyethylene composite materials, and granite are available.

The fatigue tests on the specimens were carried out following the aforementioned characteristics in fatigue machines designed for laboratory use, with cyclic loading, where the load was compressed. Each specimen is fatigued or near complete failure for about 4000 cycles. Once the specimen has been fatigued, each one is extracted and sent to the next process for obtaining computed tomography images, comprising between 800 and 1700 slices.

The specimens are placed in a laboratory-grade tomograph (phoenix v|tome|x m from General Electric), at a distance between 7 and 10 mm, as appropriate, to perform the tomography and obtain an image of each slice in high resolution: 2014 × 2024 pixels. Approximately 1700 cuts are made in the section of the specimen and processed with the definition required for the correct identification of the microfractures. To obtain correct cuts of the specimen, a power of 6 W is assigned to the tomograph; a voltage of 60 kV and a current of 100 μA are used.

**Figure 2:**
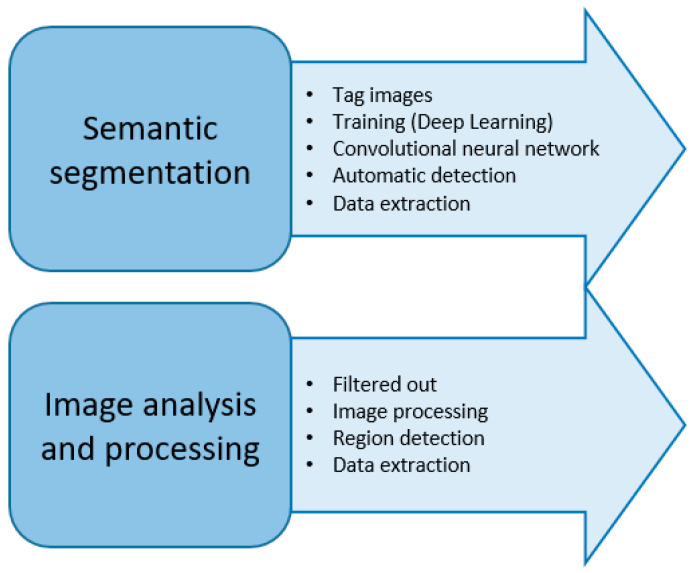
Microfracture detection methods.

Previously, the detection of microfractures in test tubes was performed individually; that is, a person analyzed each of the images, marking which and where microfractures were found. In this study, modern tools are used, such as image processing and the use of neural networks. As seen in Figure 2, there are two methods used in tandem to detect microfractures: one involves image analysis and processing, and the other uses semantic segmentation. MATLAB R2014a, with its machine learning and image processing tools, was used as the software for both cases, and codes were generated to bring everything together.

**Figure 3:**
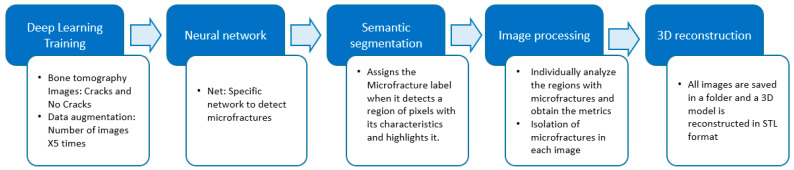
Methodology for detecting microfractures.

The route that was followed as a methodology for the detection of microfractures and their analysis is described in Figure 3, which consists of 5 main stages.

It was determined to build a new neural network with the express purpose of identifying microfractures. Said neural network should be able to read, as an input image, any resolution and scale it to a resolution of 480 × 854 or 960 × 960 pixels with 3 color channels; translated into MATLAB, a vector example [960, 960, 3] serves as the input layer or represents a color channel (grayscale). The neural network was generated, following these considerations, with 15 processing layers, in addition to integrating the SGDM (stochastic gradient descent with momentum) technique into its vectors. The neural network’s output enables us to categorize, as a first instance, the images that have microfractures and those without, dividing them into two classes.

**Figure 4:**
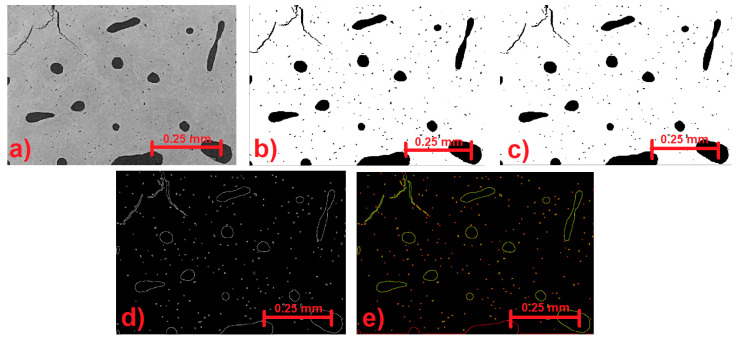
(**a**) Original CT image; (**b**) binarized image; (**c**) image after removing isolated regions and filling gaps; (**d**) image after applying a Canny filter; (**e**) detection of interior (green) and exterior (red) edges.

Due to the small database of tomography images, both with and without microfractures, the data augmentation tool was used to train the neural network. By using this method, you can artificially and considerably increase the amount of training data for the neural network. Semantic segmentation was also employed, which necessitates five steps: data extraction, automatic detection, convolutional neural networks, training (deep learning), and labeling images. One of the test images as it appeared on the tomography is shown in Figure 4a. In turn, there are various aspects of picture pre-processing, such as binaryization (converting an image to black and white), noise reduction filters, removing isolated areas, and removing porosities in materials smaller than a specific size. The code was designed to binarize the image using MATLAB tools, then remove isolated sections, fill in pixel gaps, and remove isolated regions again. Figure 4c,d provide an example of the pre-processing of the image in Figure 4a. The idea behind picture segmentation is to separate each porosity, microfracture, and feature that appears in the image by splitting the sets of pixels that indicate the shape into areas. The image was segmented using the following methodology: 

1. Use a Canny filter to identify edges (Figure 4d).

2. Use the edges (inside and exterior) to describe the regions (Figure 4c).

3. Take out the data.

By using a Canny filter, noise can be reduced by detecting changes in pixel intensity through the first Gaussian derivative. Four filters are then used to determine the gradient intensity of the image. We can then determine the edge's gradient and direction after we have these characteristics.

In addition to the analysis of the isolated microfractures from the tomography scans, the model of the test tube is reconstructed based on the images obtained with isolated microfractures, generating a 3D element that presents only the characteristics sought in this research, such as number of total microfractures, volume total microfractures, microfracture volumetric density, and microfracture properties.

The 3D model of the test tube is reconstructed using FIJI software (ImageJ 1.54d), for which the folder containing the images obtained from automatic identification is taken. It is important to generate the model and carry out a series of comparisons between automatic models and manually generated models in order to find the accuracy of the automatic identification software.

The mesoscale model of microstructure in OOF2 (object-oriented finite element analysis, version 2.3.2) software was developed from a cross-sectional image of a microstructure obtained via computed tomography. Previously, the tomography image was digitized to distinguish between the phases found in the bone cortical tissue using ImageJ software. The microstructure of the cortical bone was subdivided into interstitial lamellae and osteonal tissue. The mechanical characteristics were then attributed to the previously separated phases using OOF2. In order to ascertain the impact of the deformation caused by the variations in the mechanical characteristics of each cortical bone phase, a finite element model was finally used. The components of cortical bone, collagen, with a Young’s modulus of E = 800 MPa, and hydroxyapatite, with an E = 22.1 GPa, are the basis for the variations in mechanical properties.

**Figure 5:**
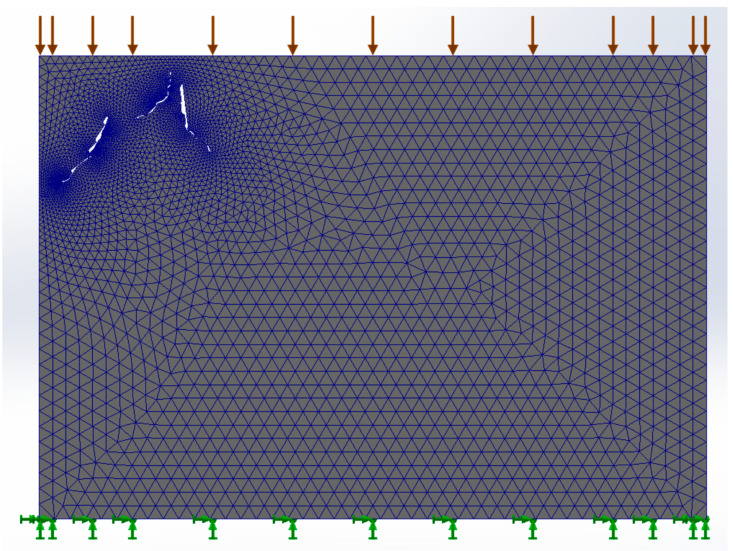
Meshing in the cortical bone structure. Boundary conditions are shown.

Figure 5 shows the meshing of the structure in the digitized image of the bone tissue. A tensile load of 100 MPa was applied (red arrows), and the movement was restricted at the bottom of the finite element model (green arrows).

As a comparison, the commonly used software SolidWorks version 28.5.0.0078 was used, which is based on the ANSYS methodology for its finite element calculations. When comparing both methods, two main factors are taken into account: processing time and the number of nodes.

**Figure 6:**
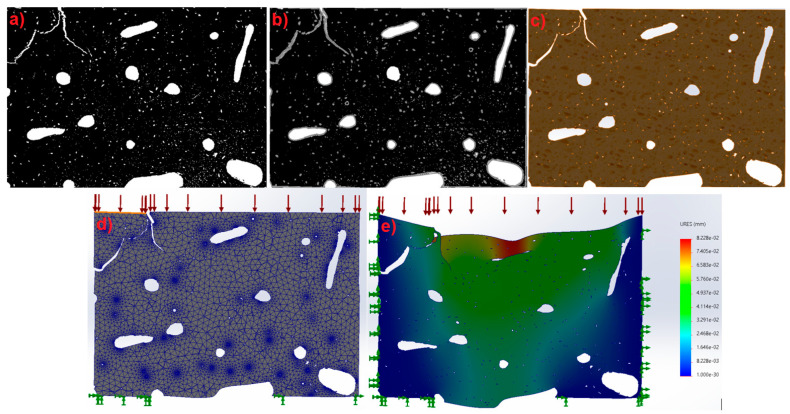
(**a**) Original binarized image; (**b**) drawing on the binarized image; (**c**) section of the digitized test tube; (**d**) meshing, loads, and fixings for the test piece section; (**e**) study carried out under the conditions of microfracture in edges.

For the mechanical simulation analysis with isolated microfracture, the resulting image from the automatic microfracture detection SolidWorks software is used, which is a binary image that includes only the microfractures and excludes the rest, as shown in Figure 6. It is important to take into account that, when the image is a square and not the actual shape of the specimen, the microfracture analysis will be affected.

## 3. Results and Discussion

For the mechanical simulation analysis involving isolated microfractures, the image resulting from the automatic microfracture detection SolidWorks software is used, which is a binary image that includes only the microfractures and excludes the rest. The image is entered into SolidWorks software to generate the drawing that simulates the microfractures. This process is performed automatically with the autotrace function. Once the figure is generated, the previously determined thickness of 2.35 μm is added, and the material (Figure 6), which is a mixture of collagen and hydroxyapatite, is described in this same section, including its mechanical characteristics. Microfractures are taken as hollow sections. The corresponding mesh is generated with the greatest possible precision using the software, which has a maximum edge dimension of 69 μm and a minimum of 1.4 μm, according to its equivalence in image pixels. The specimen is analyzed with the load equivalent to the corresponding section of 100 MPa; that is, the load is divided among the 1277 elements, using shell elements, resulting in a load of 0.078 MPa. With the highest level of initial mesh, the mesh is generated automatically. Once the fixed point and load variables have been adjusted, an analysis is carried out to obtain the results of total displacements in each axis, as well as normal, shear, and von Mises forces.

**Figure 7:**
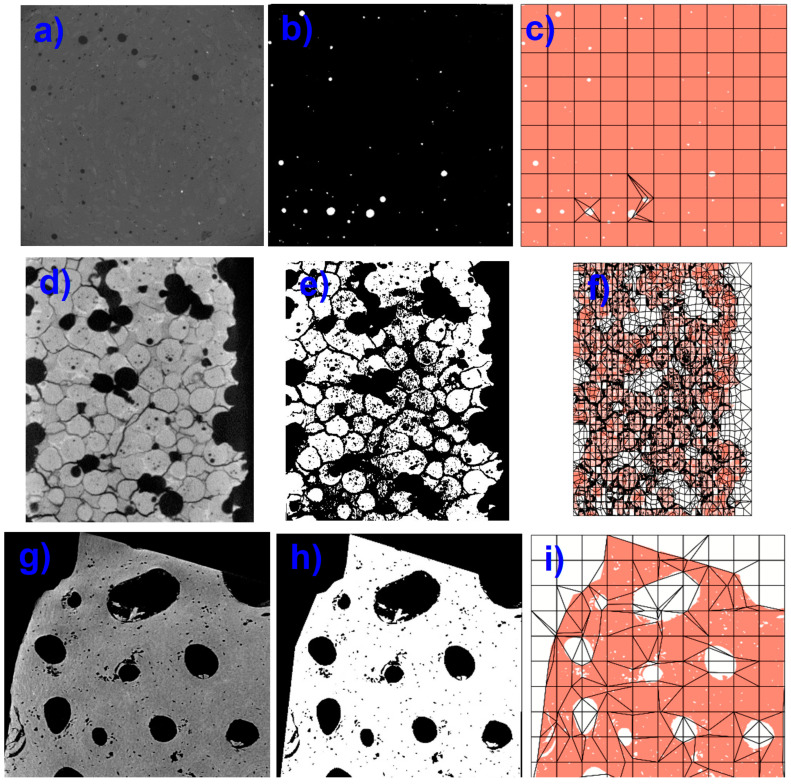
(**a–g**) Original tomography images. (**b–h**) Digitization of images for phase separation. (**c–i**) Meshing of the microstructure.

The procedure used to analyze the cortical bone microstructure is depicted in Figure 7. The original X-ray computed tomography images of bone tissue are displayed in Figure 7a,d,g, and their digitization for phase separation is shown in Figure 7b,e,h. Ultimately, using the OOF2 program, the microstructure meshes are obtained in Figure 7c,f,i.

**Figure 8:**
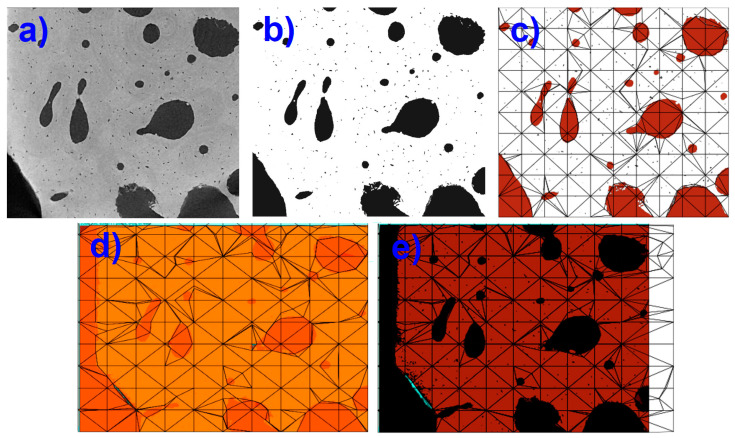
Displacement field generated in the cortical bone structure. (**a**) CT scan imagen. (**b**) Digitization of images for phase separation. (**c**) Mesh displacement. (**d**) Displacement: −8.04 mm. (**e**) Displacement: −18.09 mm.

The simulation results are shown in Figure 8. There are two phases in the microstructure of cortical bone: the ductile phase (collagen phase) and the rigid phase (hydroxyapatite phase). Its deformation becomes uneven as a result of this variation in mechanical action. The deformation field of the composite microstructure is displayed in Figure 8a,b. We also show the mesh displacement in Figure 8c and displacements at different deformations in Figure 8d,e. Collagen is more ductile in this situation than hydroxyapatite; fractures are centered in the matrix and cease to form at the osteon. A method for digitizing pictures of cortical and trabecular bone could be created. The differentiation of the current phases in the microstructure (osteons, interstitial zone, pre-cracks, and pores) is made possible using this digitization method. Furthermore, the microstructure’s meshwork might be generated, helping to create finite element models.

We will use the cohesive zone model and the extended finite element approach to forecast the initiation and propagation of cracks in these kinds of microstructures. The simulations of the specimens in SolidWorks were performed in two different ways: with uninsulated microfracture and with isolated microfracture. This methodology was used to compare the development of microfractures not only between finite element methods but also between elements that affect the material.

**Figure 9:**
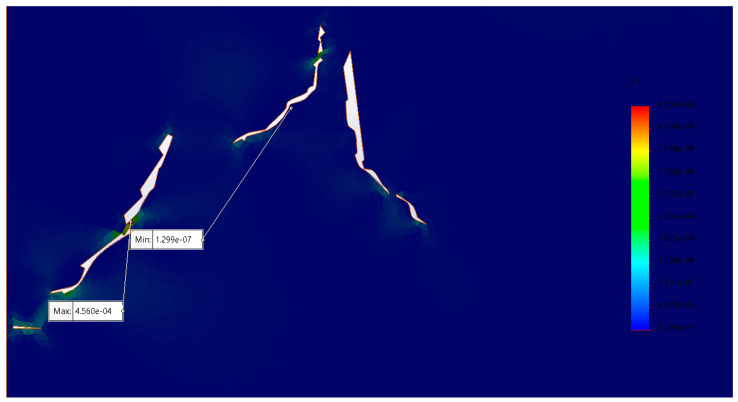
First principal stress in the region of interest of the specimen (units in MPa).

**Figure 10:**
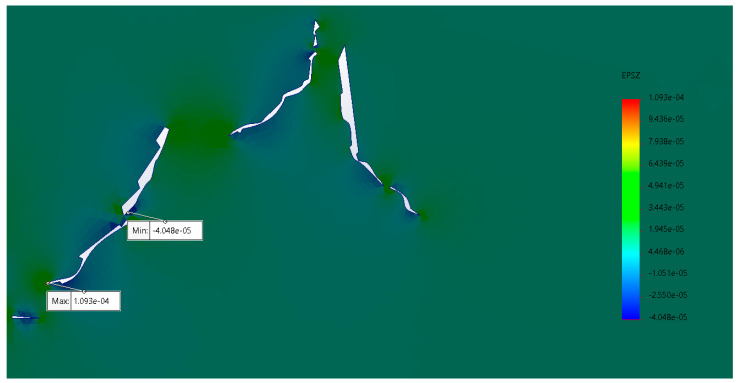
Principal stress in the Z axis; the values indicate that the material supports the main load; however, there are areas between microfractures that promote their conjunction (units in MPa).

The results of the main stresses in the microfractures in collagen and hydroxyapatite (Figure 9 and Figure 10) showed a growth trend in the areas where they were thinner. Similarly, in the areas where two microfractures converged, becoming one and generating a larger one, this led to the material fracturing completely. The principal stresses do not represent a value even close to the maximum stress supported by the material; however, being subjected to fatigue means that the energy cannot be dissipated elastically and generates microfractures. Compression stress values in the upper area of the specimen made it possible to simulate the physical fatigue analyses to which they were subjected.

**Figure 11:**
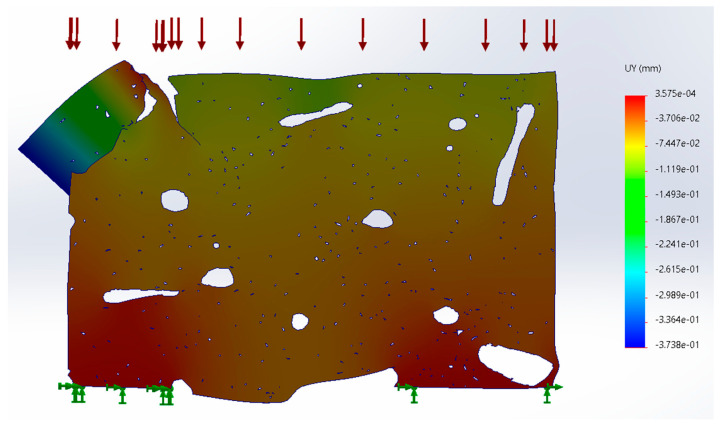
Material displacement in cross-section simulation (units in mm).

**Figure 12:**
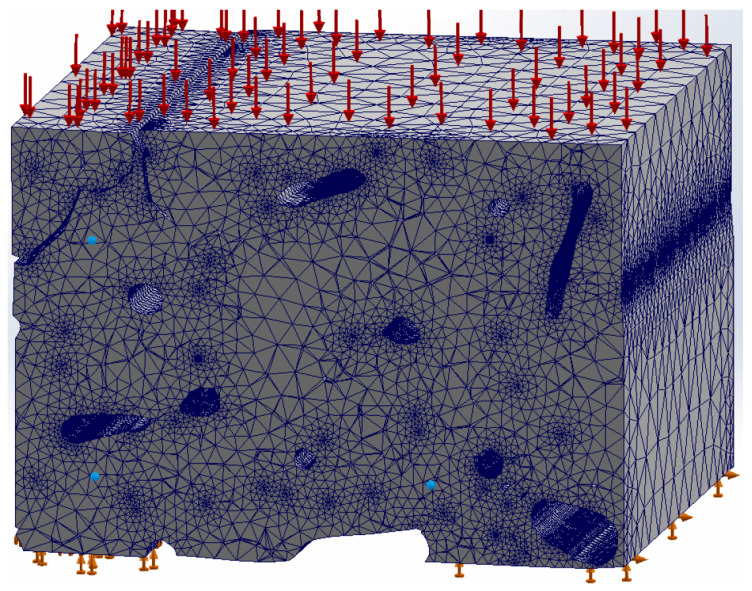
Meshing for full section simulation.

**Figure 13:**
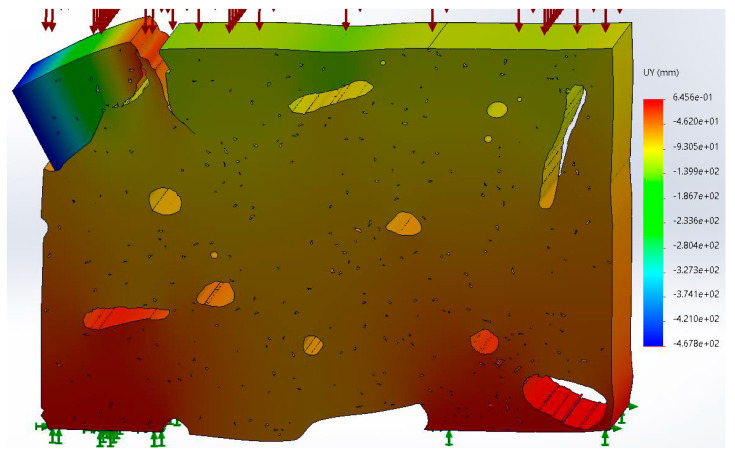
Displacement for full section simulation (units in mm).

Similar to simulation studies with isolated microfractures, the generation of the digital model of the tomography in its mechanical analysis determines the concentration of stress on the microfractures. In this case, two types of models were made: the thickness of the cross section of the tomography and the total thickness of the specimen if it were the same throughout its section (Figure 11). For the cross section of the tomography, a thickness of 2.35 μm was used (Figure 12). Being a specimen with a microfracture on the edge, this resulted in a section fracture. Furthermore, it was found that hydroxyapatite osteons absorb the main stresses in the specimen by moving through the ductile collagen tissue (Figure 13).

**Figure 14:**
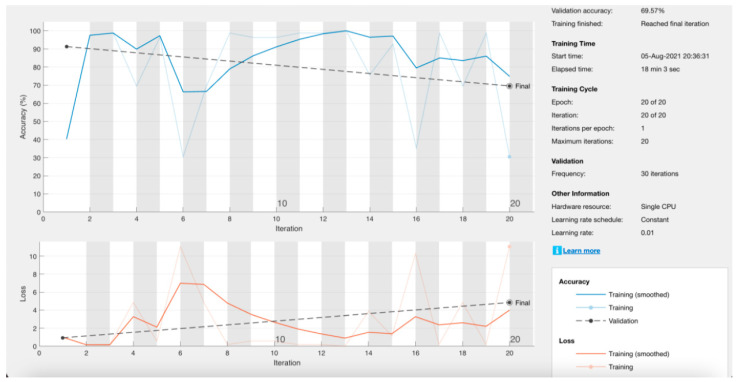
Training statistics for a neural network with a resolution of 640 × 640 pixels.

In the first instance, the neural network was designed to read 640 × 640-pixel images; however, very low precision results were obtained (Figure 14). It was decided to reduce the resolution of the image to facilitate training. The different previous stages in which the network was trained are described in Table 1.

**Figure 15:**
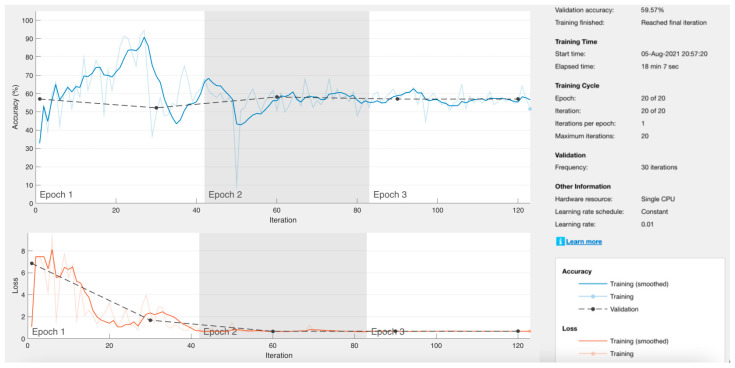
Results of training the neural network with data augmentation for images without microfractures.

Throughout the time in which this research was carried out, modifications, updates, and new training were made to the neural network. The version that worked best for the classification of microfracture images was retained. The processing time was also improved depending on the training iterations, despite the greater number of images available to train the network. There were 3028 images with microfracture and 687 without microfracture. As the last training stage, it was decided to use the data augmentation technique only for images without microfracture, thus equalizing the distribution of the classes. However, the results obtained were not satisfactory (Figure 15) and it was decided to use the previous training.

**Figure 16:**
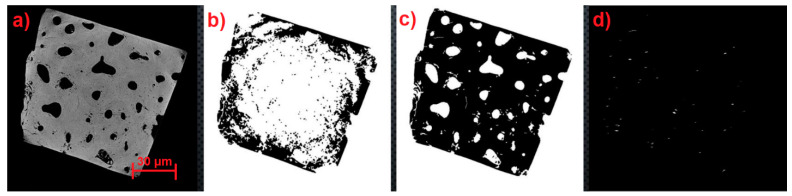
Computed tomography and its process of identifying microfractures until they are isolated. (**a**) CT image; (**b**) Binarization image; (**c**) Pore isolation image and; (**d**) Fractures isolation.

Several stages and several iterations of the detection software were generated to find a correct result of each type of material. An example is shown in Figure 16, which shows the four stages of image processing: CT image (Figure 16a), binarization (Figure 16b), pore isolation (Figure 16c), and fractures isolation (Figure 16d). Which is from a group of scans that turned out to complicate the development of the software.

**Figure 17:**
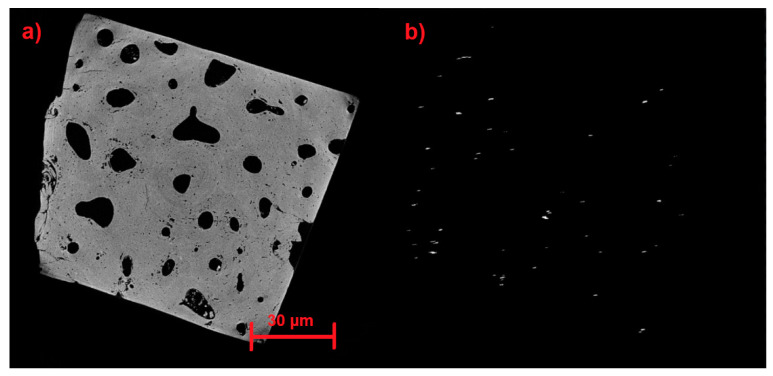
(**a**) Detection in the previous stage of automatic identification; (**b**) Unwanted items are displayed.

**Figure 18:**
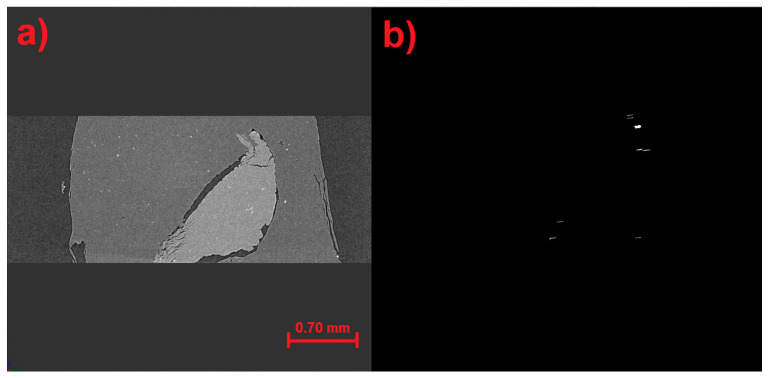
(**a**) Detection in the previous stage of automatic identification. (**b**) Elements that do not correspond to microfractures are shown.

**Figure 19:**
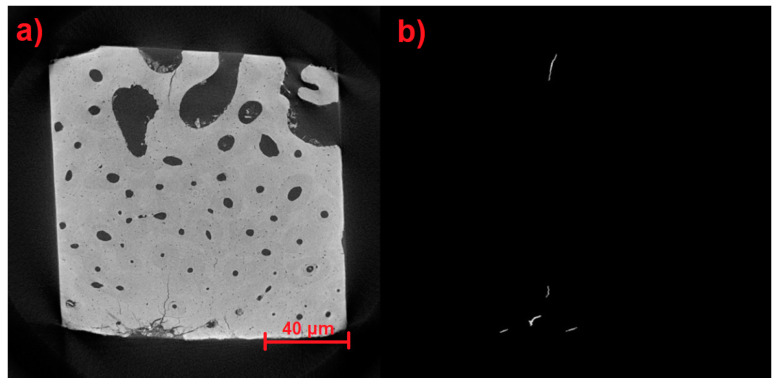
(**a**) Detection in the previous stage of automatic identification. (**b**) The largest microfractures are shown; however, the smallest ones are not detected.

Figure 17, Figure 18 and Figure 19 show the results of the identification software in its previous stages until reaching very precise results in its final stage. Figure 17a, Figure 18a and Figure 19a illustrate the detection process from the earlier automatic identification step. Figure 17b displays elements that are not wanted, Figure 18b displays elements that do not match microfractures, and Figure 19b shows the largest microfractures, however, the smallest ones are not detected.

**Figure 20:**
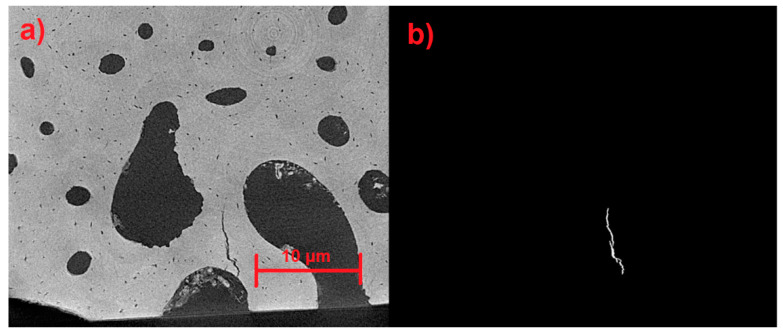
(**a**) Automatic identification current detection. (**b**) All microfractures larger than 5 pixels are detectable.

All the necessary modifications were made to the automatic detection model until reaching the current mode. In this model, the greatest number of microfractures, with their greatest extension, is identified, no unwanted elements are included, and there is a detection precision of 5 pixels, covering the total dimensions of the image. In addition, the model does not modify its morphology. Figure 20a displays a single microfracture measuring 12–13 μm in length. The microfractures in this sample region range in length from 4 to 30 μm (Figure 20b).

It is important to give the software user the possibility of changing parameters to be able to find the microfracture in the image, which is why the resolution of the tomography, the number of images to be processed, the material, and the folders where they are included as input data are crucial; images are read and saved. However, despite having powerful software, there are microfractures that cannot be detected due to their size in pixels, which is independent of the resolution in μm/pixel, due to the characteristic of circularity or eccentricity of the figure. This is a calculated parameter to a certain extent in image processing. The minimum extension to detect microfractures is 5 pixels, which translates to an extension in microns ranging from 7 μm to 20 μm, depending on the tomography. An artificial increase in the resolution of the images is proposed to facilitate their detection.

The mechanical analysis of finite elements was carried out through the comparison of ANSYS and ABAQUS methodologies, both being conclusive in studies with similar results. Due to the complexity of the research study, the mechanical simulation analyses was carried out in two different blocks. In the first instance, manual image processing for phase isolation and meshing was performed using OOF2 software. In the second part, we worked with the images obtained from the microfracture identification software, as well as the manually processed images. The objective of working with the methodology of both sections was to determine how much precision existed in the automatic identification software and the feasibility of using the resulting images for mechanical simulations.

Based on advances in the automatic identification of microfractures, the aim is to validate the precision of the neural network in identifying these microfractures in new materials that are awarded by research institutions and private companies. We are working on the identification of microfractures in materials for food use, granite, and composites with high precision, thanks to the higher resolution tomography obtained. Likewise, the mentioned materials are added to the database to feed back to the neural network.

The subsequent fatigue tests, as well as their corresponding computed tomography scans, will allow us to continue working to identify microfractures and, once detected, carry out simulations that allow us to compare their development physically, using computerized analyses. Within the simulation stage, it is necessary to continue processing images with microfractures to generate finite element analysis, vibration modes, and loads in both software, which will allow a broader perspective on the behavior of microfractures. The conclusion drawn from the identification software is of great support in obtaining the images that must be worked on within simulations, as well as in the generation of the 3D model for finite element analysis in the total volume. Following the objective of the project and the extension granted to it, the mathematical model that describes the microfractures and how they lead to a total fracture of the material must be developed. In addition, methodologies for predicting the extension of the microfracture need to be found.

## 4. Conclusions

The sequence of the methodology, as well as its parts, was constantly updated. As a main change, within the identification of microfractures, the segmentation stage was eliminated to carry out image processing based on morphological operations, which provided a shorter processing time and more precise identification results. The processing time for the automatic identification software met the overall goal of this part, which was to reduce detection time, achieving a detection accuracy of 97.7%. It was possible to identify and isolate microfractures from a series of more than 1200 images in an approximate time of 3 min, which is significantly shorter than the identification, isolation, and processing of the images manually, which can take months.

In accordance with earlier research on prediction modalities for preventing fatigue fractures in bone tissue, we worked on the automatic diagnosis of microfractures using deep learning. Finite element modeling was one of the other analyses. These investigations began with X-ray computed tomography pictures from cortical slices of bone tissue. Semantic segmentation allowed for the proper application of processing to be verified; however, in the near future, a variety of counting methods, including those for porosities, area, perimeter, and characteristic length, will be needed.

Nucleation and fracture growth prediction will be crucial for quickly implementing the process that we have also achieved for the production and differentiation of bone phases, in order to build finite element models. A methodology based on the prediction and prevention of fracture formation in bone tissue will be confirmed through a comparison with experimental results from fatigue tests and an analysis of the development of microfractures. This methodology may be helpful in the development of new clinical approaches to the osteoporosis problem. Similarly, proper biomedical material development will be feasible with respect to the ideal reinforcements under fatigue fracture resistance conditions.

These affirmations will soon align with the current data, validating a methodology aimed at accurate prediction processes for the development of microfractures and the application of non-destructive testing to prevent fatigue fractures in bone and biomedical materials. In the next stage of the research, it is intended to present results that will lead to the implementation of a mathematical model describing the behavior of microfractures. This model will generate a field of predictive study for fractures in industrial materials, as well as in inorganic materials.

## Figures and Tables

**Table 1:** Previous iterations of the neural network with their summarized values. Data obtained directly from MATLAB for approximate accuracy.

Interaction	Microfracture Entry Images	Entry Images without Microfracture	Resolution in Pixels	Processing Time	Theoretical Precision	Data Loss	Approximate Precision
1	81	36	640 × 640	18 min 03 s	69.57%	4%	70%
2	81	36	480 × 480	08 min 08 s	95.65%	0%	80%
3	301	290	480 × 480	48 min 14 s	49.15%	1%	95%
4	1505	1450	480 × 480	52 min 49 s	50.85%	0.5%	90%
5	1505	1450	480 × 480	46 min 12 s	86.55%	0%	90%

## Data Availability

The data presented in this study are available on request from the corresponding author.
